# Predicting the location of the hip joint centres, impact of age group and sex

**DOI:** 10.1038/srep37707

**Published:** 2016-11-24

**Authors:** Reiko Hara, Jennifer McGinley, Chris Briggs, Richard Baker, Morgan Sangeux

**Affiliations:** 1 Department of Physiotherapy, The University of Melbourne, Melbourne, Australia; 2 Department of Anatomy and Neuroscience, The University of Melbourne, Melbourne, Australia; 3 The Victorian Institute of Forensic Medicine, Melbourne, Australia; 4 University of Salford, Manchester, United Kingdom; 5 Hugh Williamson Gait Analysis Laboratory, Royal Children’s Hospital, Melbourne, Australia; 6 School of Engineering, The University of Melbourne, Melbourne, Australia; 7 Murdoch Childrens Research Institute, Melbourne, Australia

## Abstract

Clinical gait analysis incorporating three-dimensional motion analysis plays a key role in planning surgical treatments in people with gait disability. The position of the Hip Joint Centre (HJC) within the pelvis is thus critical to ensure accurate data interpretation. The position of the HJC is determined from regression equations based on anthropometric measurements derived from relatively small datasets. Current equations do not take sex or age into account, even though pelvis shape is known to differ between sex, and gait analysis is performed in populations with wide range of age. Three dimensional images of 157 deceased individuals (37 children, 120 skeletally matured) were collected with computed tomography. The location of the HJC within the pelvis was determined and regression equations to locate the HJC were developed using various anthropometrics predictors. We determined if accuracy improved when age and sex were introduced as variables. Statistical analysis did not support differentiating the equations according to sex. We found that age only modestly improved accuracy. We propose a range of new regression equations, derived from the largest dataset collected for this purpose to date.

Human motion analysis has broad applications in sport, workplace ergonomics and more recently in biometrics, through the recognition of individuals from their gait pattern. A common application of motion analysis in the clinical setting is gait analysis, which plays a key role in planning orthopaedics surgical treatments in persons with walking disabilities^1^,^2^.

The location of the hip joint centres (HJC) is an important part of biomechanics modelling and has repercussion in accuracy and subsequent interpretation of gait data. For example, Stagni *et al*.^3^ studied the effect of HJC mislocation on kinematics and kinetics, and showed a 3 cm error may lead to approximately 50% difference in mean flexion-extension hip moment over the gait cycle, or a delay in the cross-over from extensor to flexor moment of 26% of the gait cycle. More recently, authors investigating the effect of HJC mislocation on results from musculoskeletal modelling showed major effect on hip joint contact forces and on hip abductor muscle forces during gait^4^,^5^.

The HJC cannot be palpated and its location needs to be estimated. In the clinical gait laboratory setting, the commonly used method is to use regression equations derived from anthropometric measurements^6^. Existing regression equations estimate the location of the HJC with variable accuracy, from 1.5 cm to more than 3 cm^7^,^8^,^9^,^10^,^11^,^12^. Functional calibration^13^,^14^,^15^ was proposed as an alternative to improve accuracy and initial results obtained in healthy adult populations were encouraging^7^,^8^,^9^. However, functional calibration is challenging for those with impaired movement control or reduced range of motion and recent studies suggest it may be less accurate in clinical populations^10^,^11^,^16^. Consequently, regression equations remain the norm in the clinical setting.

Most of the existing regression equations were developed from small sample sizes, not exceeding 32 individuals, and most of them lack variation in age and or sex. Despite known differences in the shape of the pelvis according to sex and age^17^,^18^,^19^ a single equation is currently used for all subjects. The aim of this study was to investigate whether sex and/or age specific regression equations improve the accuracy in locating the HJC.

## Results

Data were collected from 157 individuals. Thirty scans were collected for each subgroup according to sex (male/female) and age group (children, adolescents and adults) with the exception of the children group for which scans were available only for 24 males and 13 females. The sample characteristics, the coordinates of the HJC and anthropometric measurements are summarized in Table 1 of the supplementary material. The age between males and females did not differ except for the children group, where females were older than males. The age difference led to taller females in this age group. In the other groups, the males tended to have larger body size (height and weight) than females. Anthropometric measurements were not statistically different between the adolescents and adults groups, except for inter-ASIS distance (p < 0.01) with minimal effect size (Cohen’s d = 0.03). Hence, only two age groups, 1: children and 2: skeletally matured (adolescents + adults) were considered in the remainder of the study.

The accuracy of our protocol to locate the centre of the femoral head visually was 1.1 mm (SD: 0.3 mm, cf. Supplementary materials). The repeatability intra-assessor (i.e. ± 1.96 x standard error of the measurement) was ±1.4, ±1.5 and ±1.8 mm for the posterior-anterior, medial-lateral, and inferior-superior directions respectively.

### Regression equations and effect of age group and sex

The best subset analysis led to leg length (LL) being the best single predictor for all HJC coordinates (Table 2). The equations with LL demonstrated higher R^2^ value and smaller prediction error than the equations with inter-ASIS distance (IA) in all components of HJC coordinates. The use of pelvic depth as a single predictor would have led to significant improvement compared to leg length for the posterior-anterior and medial-lateral directions but not for the inferior-superior direction. Although the combination of predictors improved the R^2^ of the regressions and the mean absolute prediction error from LOOCV, this was not significant at α = 0.01.

**Figure 1:**
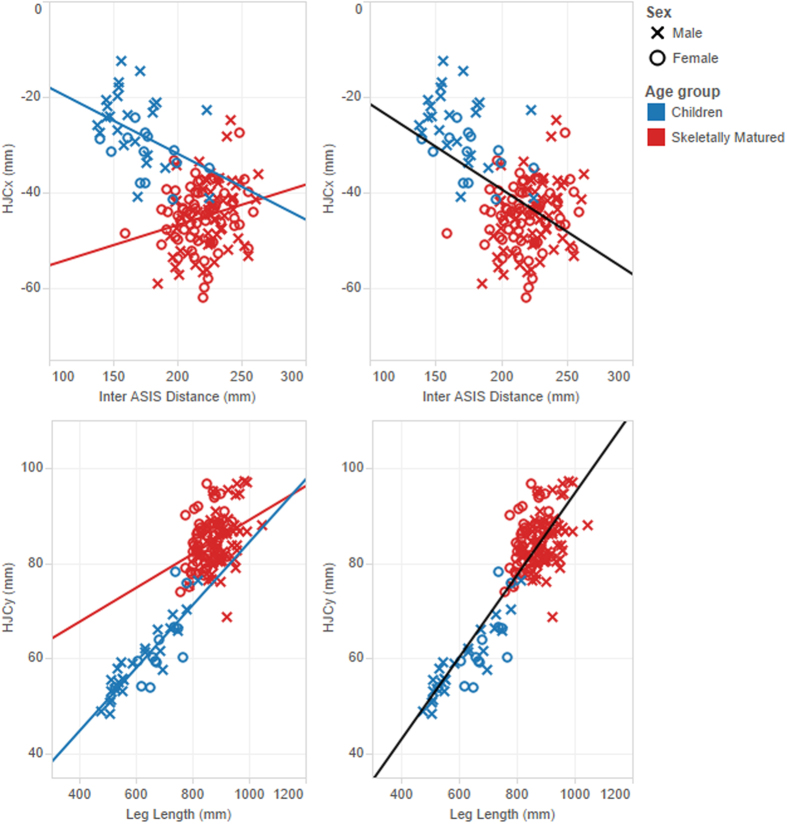
Examples of differentiating slopes according to age category: inter ASIS distance and HJC_X_ (top) and leg length and HJC_Y_ (bottom). The regression line drawn from the skeletally matured groups did not generalise (left graphs) and provides minimal or no improvement (Table 2) over the lines drawn from the combined groups (right graphs). Origin of the pelvis is the midpoint between the left and right ASIS and X: posterior-anterior direction, negative is posterior, Y: medial-lateral direction, positive is lateral, and Z: inferior-superior direction, negative is inferior.

**Figure 2:**
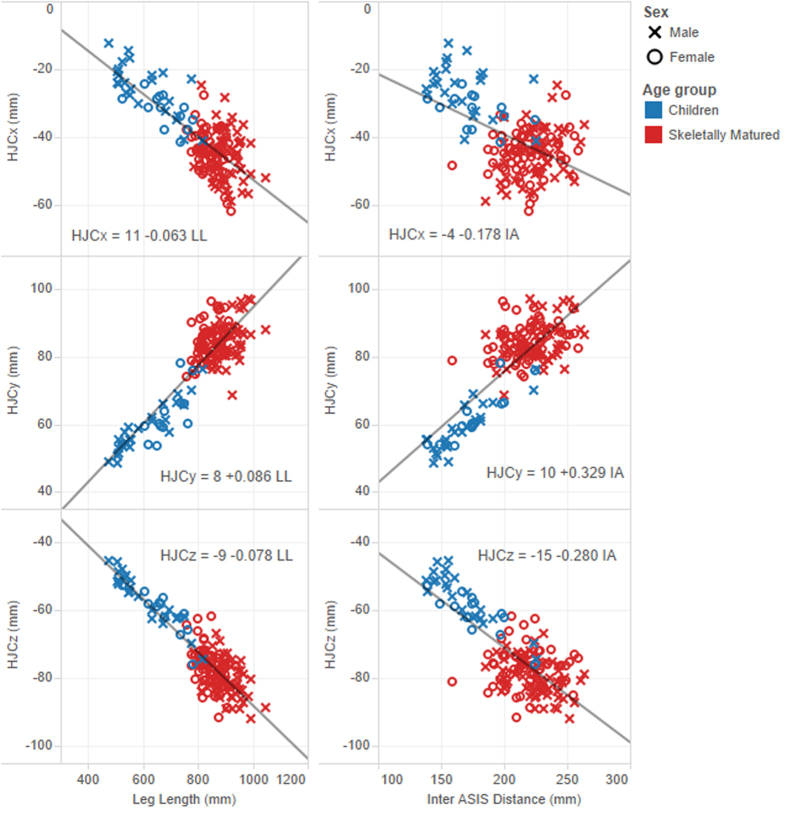
Regression equations for the hip joint centre coordinates according to leg length (left hand side) and inter ASIS distance (right hand side). Results from Table 2 indicate including age group category would not lead to significant improvements for the regression equations with leg length as single predictor (left hand side). However, significant improvements (Table 2) would be obtained for regression equations with inter ASIS distance as single predictor and different intercepts for the children and skeletally matured groups (i.e. 2 parallel regression lines, right hand side). Origin of the pelvis is the midpoint between the left and right ASIS and X: posterior-anterior direction, negative is posterior, Y: medial-lateral direction, positive is lateral, and Z: inferior-superior direction, negative is inferior.

The stepwise regression analysis identified age group as a significant categorical variable for all HJC coordinates and sex was found significant for the posterior-anterior and medial-lateral directions only. However, no interaction (i.e. different slope coefficient according to the categorical variables) was advised for any coordinate with IA and/or LL as predictor(s). Insignificant interaction between predictors and the categorical variables was confirmed visually. Indeed, Fig. 1 illustrates examples of regression equations where the slopes were allowed to change with age groups. The regression line did not fit the skeletally mature group better because the data points are clustered and do not show a clear trend. Figure 2 illustrates regression lines using a single predictor (LL or IA).

Although the stepwise regression analysis suggested different intercepts for males and females, it did not reduce the leave-one-out cross-validation (LOOCV) prediction error by more than 0.2 mm (Table 2). With leg length as predictor, using different intercepts per age groups decreased the error only for the Y coordinate, and only by tenths of a millimetre and small effect size (Cohen’s d = 0.17). The differences between groups when IA was used as a predictor were larger. Different intercepts per age groups improved the accuracy for all HJC coordinates with IA, with small to medium effect sizes: Cohen’s d = 0.27, 0.45, and 0.24, for posterior-anterior, medial-lateral, and inferior-superior directions respectively. In addition, the value of R^2^ increased from 0.25 to 0.51 for the posterior-anterior direction (Table 2).

### Generalisability of the equations

For all equations, the mean absolute prediction errors on the left side demonstrated similar values to the errors from LOOCV, with differences within 0.2 mm (Table 3, upper part). We propose the equations with leg length as single predictor for use in clinical gait analysis:


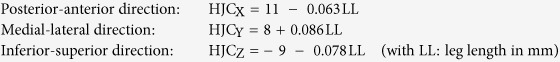


**Figure 3:**
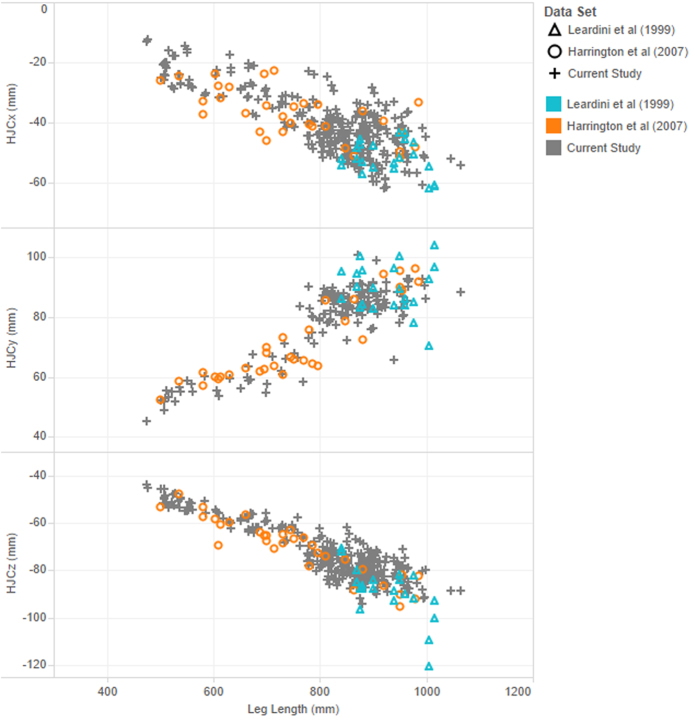
Distribution of data samples between the current and previously published studies. Samples of the current study are similarly distributed particularly with ones from Harrington *et al*.^20^. Origin of the pelvis is the midpoint between the left and right ASIS and X: posterior-anterior direction, negative is posterior, Y: medial-lateral direction, positive is lateral, and Z: inferior-superior direction, negative is inferior.

The results from Harrington *et al*.^20^ presented similar errors compared to these equations while the errors were slightly larger with the data from Leardini *et al*.^7^ (Table 3, upper part). The mean absolute prediction error from the equations of the current study was comparable to Harrington *et al*.^20^ and considerably smaller than that from Bell *et al*.^21^,^22^ or Davis *et al*.^23^ (Table 3, lower part). Figure 3 highlights similarities of the data available from Leardini *et al*.^7^, Harrington *et al*.^20^ and the current study.

## Discussion

Although the pelvis is known to have considerable morphological differences between sex^19^,^24^, the results of our study showed the position of the HJC in the pelvic anatomical coordinate system was not one of these major differences. Leg length was found to be the best predictor for all coordinates and our analysis did not support the use of additional anthropometric (inter ASIS distance) or categorical (age, sex) predictors. Using leg length as the single predictor, the accuracy of the regression equations to locate the HJC was 5.2 mm, 4.4 mm, and 3.8 mm in the posterior-anterior, medial-lateral and inferior-superior directions respectively (Table 3). Although inter ASIS distance is a commonly used predictor, the equations with inter ASIS distance as predictor did not perform as well as those using leg length.

For all coordinates, differentiating the rate of change with the predictors (leg length or inter ASIS distance) according to age groups did not improve the prediction accuracy statistically. The scatter plots on Fig. 1, in particular for the posterior-anterior direction (X), shows the trend is weaker when only data from skeletally mature individuals are used (especially with inter ASIS distance) and does not generalize well. By utilizing data from all age groups, the range of values increases for both the dependent and independent variables, and the trend is more representative for the overall group. Most studies prior to Harrington *et al*.^20^ determined the regression equations based on adult data only and these performed less well in children^10^. Our results confirm that anthropometric regression equations require the widest possible range of sizes and skeletal maturity level to generalize.

The results indicate that using different intercepts for age groups improves the prediction accuracy for the medial-lateral direction (Y) only, when using leg length as the anthropometric variable. That may be explained by the continued growth of the segments in the lateral direction, well after longitudinal growth of long bones ceased^25^. However, differentiating intercepts by age groups in this case led to small improvements (Cohen’s d = 0.17). Overall, our results do not suggest the equations with leg length need to change according to sex or age group (Fig. 2).

Differentiating intercepts according to age group might have a greater influence for equations with inter ASIS distance as demonstrated by the reduction of the mean prediction error: 1.3, 1.7, 1.0 mm for the posterior-anterior, medial-lateral, and inferior-superior directions, respectively. It is important to note that regression equations using inter ASIS distance as predictor may still be preferable for individuals with conditions in which leg length does not reflect the overall anatomy, such as in dwarf syndrome.

The generalizability of the equations, obtained from data on the right side, was tested using leave one out cross validation and prediction accuracy compared to data from the left side. Both tests presented equivalent values of prediction error. Our equations also demonstrated comparable accuracy when applied to an independent data set, (Table 3, upper-part, Harrington *et al*.^20^). Slightly larger errors were found when our equations was applied to data only including adult subjects (Table 3, upper-part, Leardini *et al*.^7^).

Improved accuracy of our equations was found when compared to previously published equations, except to those using pelvic depth as predictor (Table 3, lower-part). It is important to note that accuracy in this study, and in previous studies^20^, was calculated from medical imaging using the true pelvic depth. The accuracy of regression equations using pelvic depth may not translate to the clinical setting because of the thickness of soft tissues over the ASI and PSI bony landmarks. Soft tissues over the ASIs and PSIs affect the clinical measurement of pelvic depth and Sangeux^12^ found measurements of pelvic depth were systematically larger in the clinical setting compared to medical imaging, whereas inter ASIS distance and leg length measurements were not. Systematic bias was found when pelvic depth was used as a predictor for the posterior-anterior and medial-lateral directions^12^.

It is important to note that soft tissue thickness over the ASIs affects the origin of the pelvis anatomical coordinate system in the posterior-anterior direction and therefore the accuracy of all equations^12^. Consequently, a solution to measure the thickness of soft tissue over the ASI bony landmarks during static standing calibration appears as the next logical step to improve the localisation of the HJC from regression equations.

Selection criteria in our study excluded subjects with known musculoskeletal problems to avoid including abnormal proportions in the anthropometric predictors. Therefore, our results have uncertain ability to generalize to individuals with pathology or gait impairment and may still require validation for specific clinical applications. However, it is worth noting that Harrington *et al*.^20^ found no difference in the HJC prediction error between typically developed children and children with cerebral palsy, using a range of equations including theirs and that of Bell *et al*.^21^,^22^, Davis *et al*.^23^, and Orthotrak^20^.

A limitation of our study is the restricted details of the subjects. For example, the health and nutritional status, physical activity level or onset of puberty within the sample was not controlled nor known due to the nature of the data. These factors may have influenced bone development or structure. Similarly, race was not determined. We found similar relationships in the data between the position of the hip joint centres and inter ASIS distance, leg length, and pelvic depth from a variety of samples drawn from different countries (England^20^, Italy^7^, and Australia: current study, Fig. 3). However, the Caucasian race may be the most prevalent in these countries and our results may need to be confirmed with samples including different races.

## Material and Methods

### Study materials

Approval was granted by the custodian of the medical images, the Ethics Committee at the Victorian Institute of Forensic Medicine (VIFM, ref#: EC 15/2012) and the University of Melbourne. All methods were performed in accordance with the relevant guidelines and regulations. De-identified full-body cadaveric CT scans from individuals of different ages and sex were reviewed. All CT scans were collected between January 2007 and May 2013 as part of routine procedures. Images were taken every 1.0 mm with a slice thickness of 1.5 mm in all planes, leading to 33% overlap between slices to improve 3D display and multi-planar reconstruction^26^.

A sample of 180 scans, 30 scans in each of 6 groups: 3 age groups of both sex, was sought for collection. The age groups were classified as 1) children, 2) adolescents, and 3) adults, and consisted of individuals aged 5–11, 16–19, and 25–40 years old, respectively. Children aged between 12 and 15 were excluded since individuals in these ages would likely be in the middle of puberty during which peak growth in height is most likely to occur^27^. Subjects older than 40 years old were not included to avoid including persons with age-related degeneration of bones and joints.

To maximize the likelihood of attaining ‘typically developed’ or ‘healthy’ samples, exclusion criteria were: a) known trauma to pelvis and/or lower extremity, b) known prior musculoskeletal disease or conditions which caused obvious changes in bony structure, or c) known growth or developmental disorders affecting growth or musculoskeletal structure (e.g. Down’s syndrome, cerebral palsy, myelomeningocele, arthrogryposis, Dravet’s syndrome, osteoarthritis, etc.).

In addition to CT-scans, sex, age, height, weight, and cause of death were obtained. Sample characteristics were compared using descriptive statistics and two-sample t-tests.

### Data processing

On each scan, the locations of 12 anatomical landmarks (Table 1) were identified by visual inspection from a single assessor (RH) using 3D Slicer (http://www.slicer.org). The method for localisation of the centre of the femoral head, assumed to be the HJC, followed an established protocol to determine femoral neck anteversion and neck shaft angle^28^. Coordinates of the landmarks were exported to calculate inter ASIS distance (IA), leg length (LL), pelvic depth (PD) and total pelvic width (TPW, Table 1). Although used in some equations in prior studies^21^,^29^,^30^, the pubic symphysis landmark and related measurements were not included in the current study due to the difficulty in identification of these landmarks in routine clinical examinations.

The position and orientation of the pelvis was defined according to the conventional gait model^23^. Coordinates of the HJC were determined in relation to the pelvic origin: the midpoint between the left and right ASIS with the HJC posterior, lateral and inferior compared to the origin. The X coordinate (posterior-anterior direction) was negative when posterior to the origin, the Y coordinate (medial-lateral direction) was positive when lateral to the origin and the Z coordinate (inferior-superior direction) was negative when inferior to the origin.

To estimate the accuracy of localisation of the HJC^28^, data from 10 subjects (20 limbs) selected at random were compared with automatic fitting of a sphere to 3D shape model of the femoral heads (ref. 31, details in supplementary material). To assess intra-assessor repeatability, identification of all landmarks was repeated three times on the same 10 subjects (20 limbs). Repeatability was assessed for the X, Y and Z coordinates of the right and left HJC and we calculated the standard error of the measurement (SEM).

### Generation of equations

Regression equations were developed to estimate the location of the HJC from linear combinations of predictors with intercept. Data from the right side of each subject was utilized to generate equations and the best predictors were identified from a best subset regression analysis (Minitab 17, Minitab Inc., USA) between the predictors IA and LL. Pelvic depth was not included in the best subset regression analysis as the purpose was to develop equations with clinical utility. Although pelvic depth, derived from medical images, may be a good predictor for the position of the HJC^20^, the presence of skin and adipose tissue between the skin surface and the bony landmarks introduces an unknown magnitude of error in the corresponding clinical examination measurement and, as a consequence, in the estimate of the location of the HJC^12^. Regression equations including PD were derived and provided for comparison purposes only. The subjects’ age was also not included in the regression model since age did not display a linear trend with respect to the response variables across groups (cf. Fig. 1 in Supplementary Material) and age was kept as a categorical variable (1: children, 2: adolescents and 3: adults).

For each equation, the goodness of fit was estimated with the coefficient of determination (R^2^) of the multiple linear regression and the prediction accuracy by the mean absolute error from leave-one-out cross-validation (LOOCV)^20^,^32^. LOOCV provides a means to estimate the prediction accuracy of the regression equations for new samples. It is a particular case of k-fold cross-validation when k = 1. Given n data points, LOOCV derives n regression equations each based on n-1 data points, hence for each equation, one data point has been removed from the dataset and the absolute difference between the data point value and its estimate from the regression equation provides the prediction error.

To determine whether equations with increasing numbers of predictors led to significant improvement, the absolute prediction errors from the equations with increasing number of predictors were compared to those of the single predictor equation with paired t-test at α = 0.01. A stepwise regression analysis was also performed to investigate whether the two categorical variables, age group and sex, may be considered in the regression model. The model included the predictors, the categorical variables and the interactions between predictors and categorical variables.

### Generalisability of the equations

To examine the generalisability of the equations, we applied them to the data from the left side, which was not included in the development of equations, and computed the mean absolute errors (MAE) for each subject. The generalisability of the equations was further evaluated, using data sets available from previously published studies (Harrington *et al*.^20^ and Leardini *et al*.^7^. The accuracy of equations derived in this study was also compared with common regression equations that have previously been published, e.g. Bell *et al*.^22^, Davis *et al*.^23^, and Harrington *et al*.^12^,^20^.

Statistical analysis was performed in Matlab with the statistics and machine learning toolbox (version 10.1, The Mathworks, USA).

## Additional Information

**How to cite this article**: Hara, R. *et al*. Predicting the location of the hip joint centres, impact of age group and sex. *Sci. Rep.* **6**, 37707; doi: 10.1038/srep37707 (2016).

**Publisher's note:** Springer Nature remains neutral with regard to jurisdictional claims in published maps and institutional affiliations.

## Supplementary Material

Supplementary Data 1

## Figures and Tables

**Table 1:** Bony landmarks identified and anthropometric measurements collected.

	Name	Abbreviation	Description/Definition
Bony Landmark	Iliac crest	IC	Most lateral point of the pelvis
	Anterior superior iliac spine	ASI	Most anterior point of the pelvis
	Posterior superior iliac spine	PSI	Most posterior point of the pelvis
	Hip joint centre	HJC	Centre of the femoral head (visualized in three planes on CT scans^28^)
	Medial epicondyle of femur	ME	Most medial point of the medial epicondyle
	Medial malleolus	ML	Most prominent point of the medial malleolus
Anthropometric Measurement	Inter ASIS distance	IA	Distance between left and right ASIS
	Total pelvic width	TPW	Widest distance of the pelvis in the medial-lateral direction between ilia
	Pelvic depth	PD	Distance from the midpoint of two ASIS to the midpoint of two PSIS
	Clinical leg length	LL	Distance from ASIS to medial malleolus through the medial epicondyle of the femur

Note: all bony landmarks and clinical leg length are identified/measured bilaterally (e.g. LIC: left iliac crest, RIC: right iliac crest). The term ‘inter ASIS distance’ is used to describe the distance between two ASIS although some literature use ‘pelvic width’ to describe the measurement. ‘Total pelvic width’ indicates the maximum distance between ilia. Please cf. Fig. 2 in supplementary material for a schematic of the landmarks in 3D slicer.

**Table 2:** Comparison of regression equations with different predictor and category.

	Predictor(s)	Category	Equation	LOOCV (mm)	R^2^	Cohen’s *d*
**Posterior-Anterior direction HJC**_**X**_	IA	none	−4 −0.178 IA	6.9	0.25	**(Ref a)**
		sex	−2 −0.176 IA −3.340 sex	6.8	0.27	NS^a^
		age	−30 +0.014 IA −17.964 age	5.6	0.51	0.27^a^
		sex + age	−29 +0.009 IA −1.777 sex −17.481 age	5.6	0.51	0.28^a^
	LL	none	11 −0.063 LL	5.2	0.58	**(Ref b)**, 0.36^a^
		sex	12 −0.063 LL −3.739 sex	5.1	0.61	NS^b^
		age	−0 −0.044 LL −6.715 age	5.1	0.60	NS^b^
		sex + age	4 −0.049 LL −3.172 sex −5.062 age	5.0	0.62	NS^b^
	IA and LL	none	5 +0.112 IA −0.085 LL	5.1	0.61	0.40^a^, NS^b^
	PD	none	5− 0.353 PD	4.2	0.72	0.61^a^, 0.28^b^
**Medial-Lateral direction HJC**_**Y**_	IA	none	10 +0.329 IA	5.8	0.65	**(Ref a)**
		sex	9 +0.328 IA +2.895 sex	5.7	0.66	NS^a^
		age	34 +0.151 IA +16.714 age	4.1	0.81	0.45^a^
		sex + age	33 +0.154 IA +1.435 sex +16.324 age	4.1	0.81	0.45^a^
	LL	none	8 +0.086 LL	4.4	0.77	**(Ref b)**, 0.34^a^
		sex	7 +0.086 LL +3.588 sex	4.3	0.79	NS^b^
		age	28 +0.051 LL +12.200 age	3.9	0.83	0.17^b^
		sex + age	25 +0.954 LL +2.359 sex +10.971 age	3.8	0.83	0.19^b^
	IA and LL	none	3 +0.109 IA +0.065 LL	4.3	0.79	0.39^a^, NS^b^
	PD	none	19 +0.459 PD	3.5	0.87	0.67^a^, 0.31^b^
**Inferior-Superior direction HJC**_**Z**_	IA	none	−15 −0.280 IA	5.6	0.58	**(Ref a)**
		sex	−15 −0.280 IA −0.167 sex	5.6	0.57	NS^a^
		age	−35 −0.133 IA −13.759 age	4.6	0.71	0.24^a^
		sex + age	−36 −0.130 IA +1.090 sex −14.056 age	4.6	0.71	0.24^a^
	LL	none	−9 −0.078 LL	3.8	0.80	**(Ref b)**, 0.48^a^
		sex	−9 −0.078 LL −0.766 sex	3.8	0.80	NS^b^
		age	−18 −0.063 LL −5.403 age	3.7	0.81	NS^b^
		sex + age	−18 −0.063 LL −0.170 sex −5.315 age	3.7	0.81	NS^b^
	IA and LL	none	−8 −0.038 IA −0.071 LL	3.8	0.80	0.49^a^, NS^b^
	PD	none	−24 −0.380 PD	4.3	0.74	0.32^a^, NS^b^

LOOCV: mean absolute error from the leave one out cross validation, HJC_X_, HJC_Y_, and HIC_Z_: components of the hip joint centre, IA: inter ASIS distance, LL: leg length, PD: pelvic depth, Ref a: reference equation a = equation of IA with no category, Ref b: reference equation b = equation with LL with no category.

^a^Effect size in relation to reference equation a.

^b^Effect size in relation to reference equation b, NS: not significant difference compared to the reference.

**Table 3:** Generalizability assessment of study equations (upper part) and comparison of the study equations with previously published equations (lower part).

Equation Applied	Data Set Used	Mean absolute error (mm) and Predictor(s)					
		HJC_X_		HJC_Y_		HJC_Z_	
**Current Study**	**Current Study (Right)**	**5.2**	**LL**	**4.4**	**LL**	**3.8**	**LL**
Current Study#	Current Study (Left)	5.3	LL	4.6	LL	4.0	LL
	Leardini *et al*.^7^ (Bilateral)	6.1	LL	7.6	LL	8.7	LL
	Harrington *et al*.^20^ (Right)	5.6	LL	4.3	LL	4.0	LL
Bell *et al*.^21^	Current Study (Right)	8.1	IA	6.1	IA	10.9	IA
Bell *et al*.^22^		6.8	IA	6.1	IA	10.9	IA
Davis *et al*.^23^		9.7	LL*	12.2	LL, IA	11.2	LL*
Harrington *et al*.^20^ ^a^		4.7~	PD	5.9	IA	5.5	IA
Harrington *et al*.^20^ ^b^		4.7~	PD	3.3~	PD, IA	4.0	IA, LL

The mean absolute error for the first line (current study, right side) was obtained from the leave one out cross validation (LOOCV).

HJC_X_, HJC_Y_, and HIC_Z_: components of the hip joint centre, LL: leg length, IA: inter ASIS distance, PD: pelvic depth.

^#^Current study equations are the equations with LL with no category: HJC_X_ = 11 −0.063 LL, HJC_Y_ = 8 +0.086 LL, and HJC_Z_ = −9 −0.078 LL.

^*^The distance from ASIS to greater trochanter required for the equation was calculated from a formula, ASIS to trochanter distance = 0.1288 LL - 48.56 (*Plug-in Gait Manual*, Vicon), as the measurement was not collected from CT scans.

^a^Proposed equations with single predictor.

^b^Equations generated with the best predictor(s).

^~^Increased error is expected when pelvic depth is measured without medical images, due to the presence of skin and adipose tissue between the skin surface and the bony landmarks^12^.
