# Supplementary material for: Predicting the location of the hip joint centres, impact of age group and sex
Source: Sci Rep. 2016 Nov 24;6:37707. doi: 10.1038/srep37707 (PMC5121588; doi:10.1038/srep37707)
Supplement: Supplementary Data 1 [file srep37707-s1.pdf]

Supplementary materials to:

Predicting the location of the hip joint centres, impact of age group and sex

Reiko Hara[1], Jennifer McGinley[1], Chris Briggs[2, 3], Richard Baker[4], Morgan Sangeux[5, 6, 7, *]

[1] Department of Physiotherapy, The University of Melbourne, Melbourne, Australia
[2] Department of Anatomy and Neuroscience, The University of Melbourne, Melbourne, Australia
[3] The Victorian Institute of Forensic Medicine, Melbourne, Australia
[4] University of Salford, Manchester, United Kingdom
[5] Hugh Williamson Gait Analysis Laboratory, Royal Children's Hospital, Melbourne, Australia
[6] School of Engineering, The University of Melbourne, Melbourne, Australia
[7] Murdoch Childrens Research Institute, Melbourne, Australia

*Corresponding Author:

Morgan Sangeux
Hugh Williamson Gait Analysis Laboratory
The Royal Children's Hospital, 50 Flemington Road
3052, Parkville, Australia
Email: morgan.sangeux@gmail.com

Table 1: Sample characteristics
Hip joint centre coordinates and anthropometric measurements

Group	N	Mean (SD)			Mean (SD) (mm)						
		Age (years)	Weight (kg)	Height (cm)	HJC_X	HJC_Y	HJC_Z	IA	TPW	PD	LL
**All**	**157**	**21 (10)**	**62 (24)**	**158 (21)**	**-41 (10)**	**79 (12)**	**-74 (11)**	**209 (29)**	**249 (36)**	**130 (24)**	**818 (123)**
M	84	20 (10)	63 (27)	159 (25)	-39 (11)	77 (13)	-73 (12)	209 (33)	246 (41)	127 (28)	819 (152)
F	73	22 (10)	62 (20)	158 (15)	-43 (8)	81 (10)	-74 (9)	210 (25)	252 (30)	134 (19)	816 (79)
1	37	7 (2)	33 (16)	127 (18)	-28 (7)	60 (7)	-58 (7)	170 (23)	194 (25)	91 (14)	633 (99)
M1	24	7 (2)	30 (16)	123 (19)	-25 (8)	59 (7)	-56 (7)	167 (23)	189 (26)	87 (13)	606 (102)
F1	13	9 (2)	38 (18)	135 (13)	-32 (5)	62 (7)	-60 (6)	177 (22)	202 (23)	97 (12)	682 (72)
2	60	18 (1)	66 (14)	168 (7)	-45 (7)	83 (4)	-79 (5)	216 (18)	260 (17)	142 (8)	872 (46)
M2	30	18 (1)	71 (14)	172 (5)	-45 (8)	83 (5)	-80 (5)	221 (17)	264 (15)	141 (9)	897 (40)
F2	30	18 (1)	61 (12)	163 (6)	-46 (6)	83 (4)	-78 (5)	211 (18)	255 (17)	142 (7)	847 (37)
3	60	32 (5)	77 (19)	168 (12)	-45 (6)	87 (5)	-79 (6)	227 (18)	272 (16)	144 (10)	877 (63)
M3	30	31 (5)	82 (19)	175 (8)	-45 (7)	87 (5)	-81 (8)	230 (16)	273 (17)	145 (10)	910 (61)
F3	30	33 (5)	72 (19)	162 (11)	-45 (6)	87 (6)	-76 (6)	224 (18)	270 (15)	143 (10)	845 (46)

M: male, F: female, 1: children (5-11 years old), 2: adolescents (16-19 years old), 3: adults (25-40 years old), HJC_X, HJC_Y, and HIC_Z: components of the hip joint center, IA: inter ASIS distance, TPW: total pelvic width, PD: pelvic depth, LL: leg length. Note: HJC and LL are the mean of bilateral data.

Figure 1: Hip joint centre coordinates as a function of age

The scatter plot shows that the relation between the coordinates of HJC and age was not linear and that HJC coordinates have stabilised from the adolescent group (age > 16).

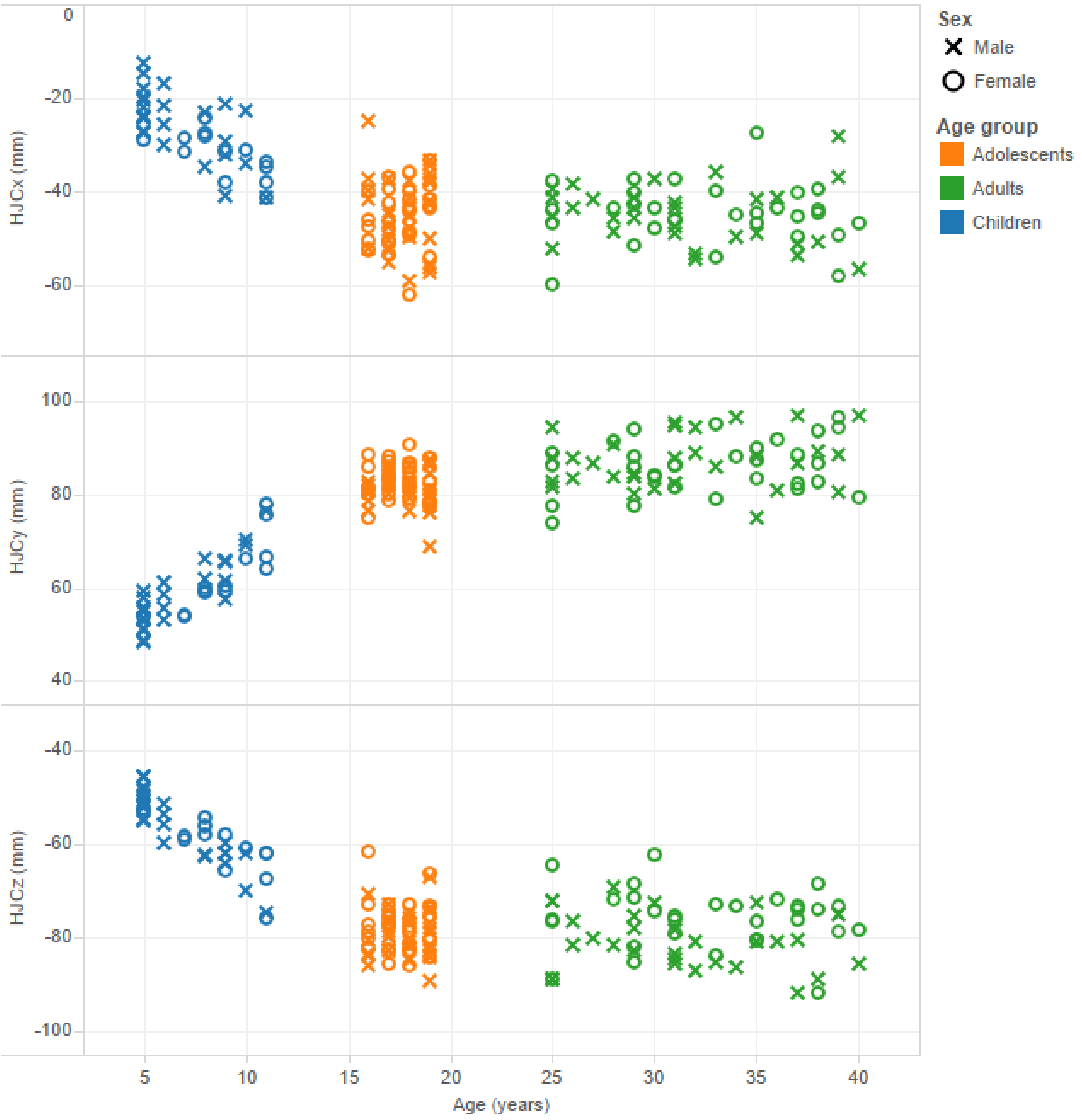

Figure 2: Identification of the landmarks in CT-scans

3D view in 3D slicer and 3 axial slices of a single subject illustrating the positions for HJC, ME and ML on the right side. Additional landmarks on the pelvis (ASI, PSI, IC) are visible in the 3D view.

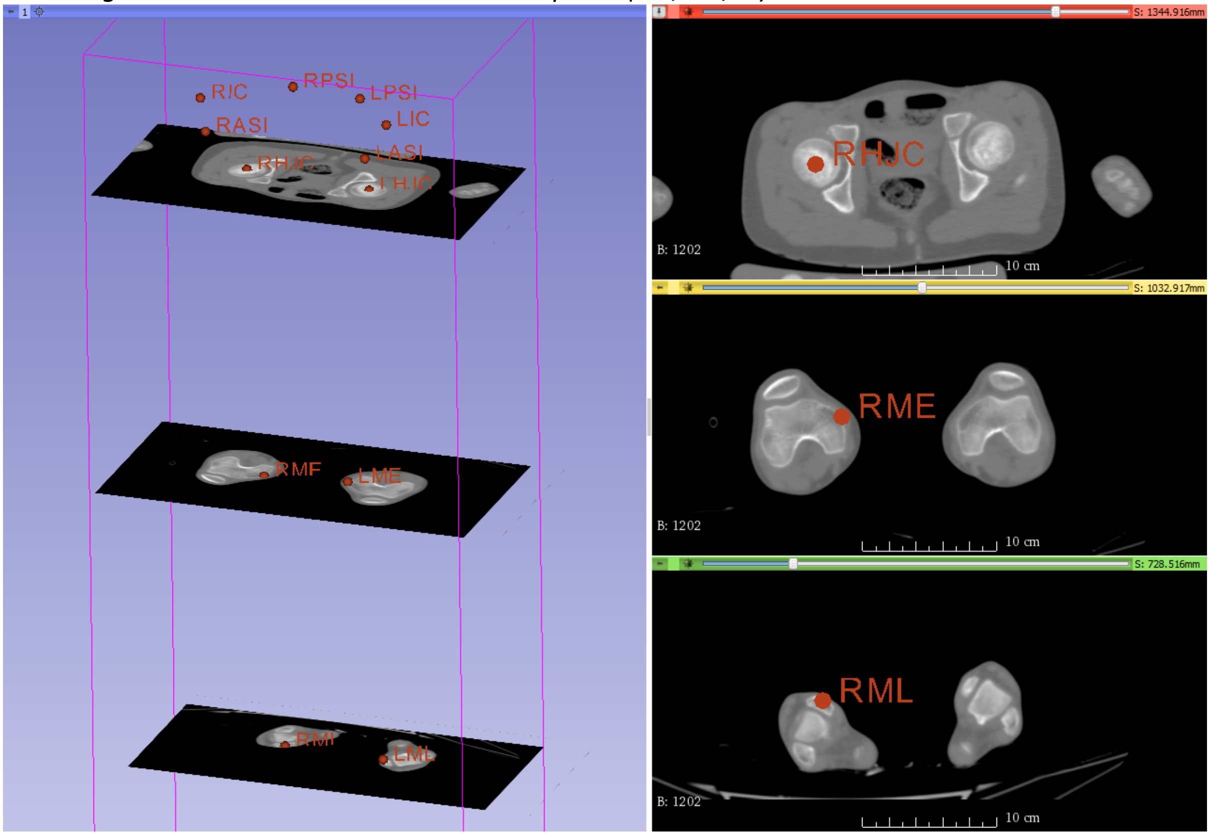

Accuracy of visual determination of the Hip Joint Centre

Three-D shape models of the proximal femurs were created in 3D slicer. The proximal femur was outlined in all slices containing the femur head and neck (Figure 3). A shape model was created from the outlines and the mesh was exported to CloudCompare (http://www.cloudcompare.org/) where a sphere was fitted to the point cloud corresponding to the femoral head[1] (Figure 4).

The femoral head centre identified from sphere fitting was compared to the repeated visual localisation (3 repeats) performed in 10 subjects (20 limbs) according to a protocol designed to determine femoral neck anteversion and neck shaft angle[2]. The results for each coordinates and 3D distance are presented in Figure 5. The accuracy of visual localisation of the femoral head compared to sphere fitting was 1.1mm (SD: 0.3mm).

Figure 3: Creating 3D models of the femoral head in 3D slicer

The top panel is the 3D visualisation window while the three sections in the bottom panel correspond to the axial (red), sagittal (yellow) and frontal (green) slice visualisation windows.

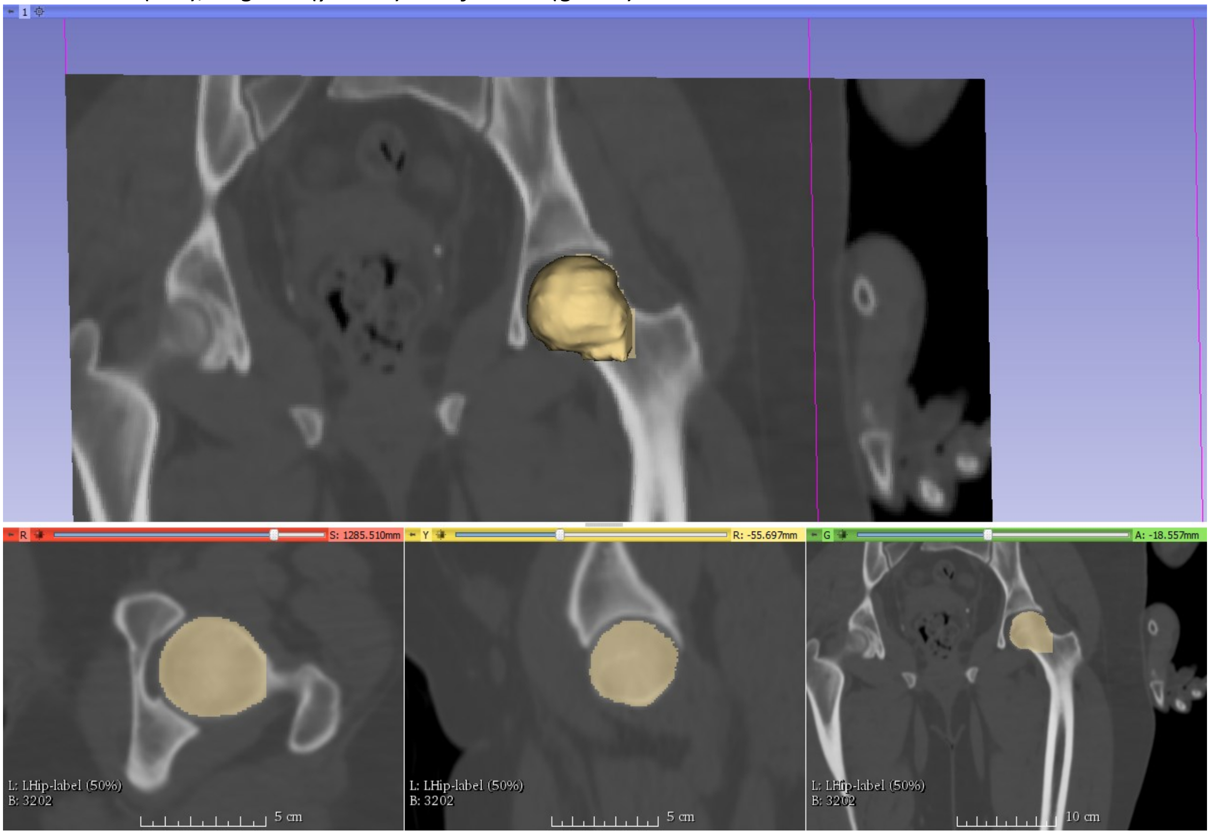

Figure 4: Fitting a sphere to the femoral head model

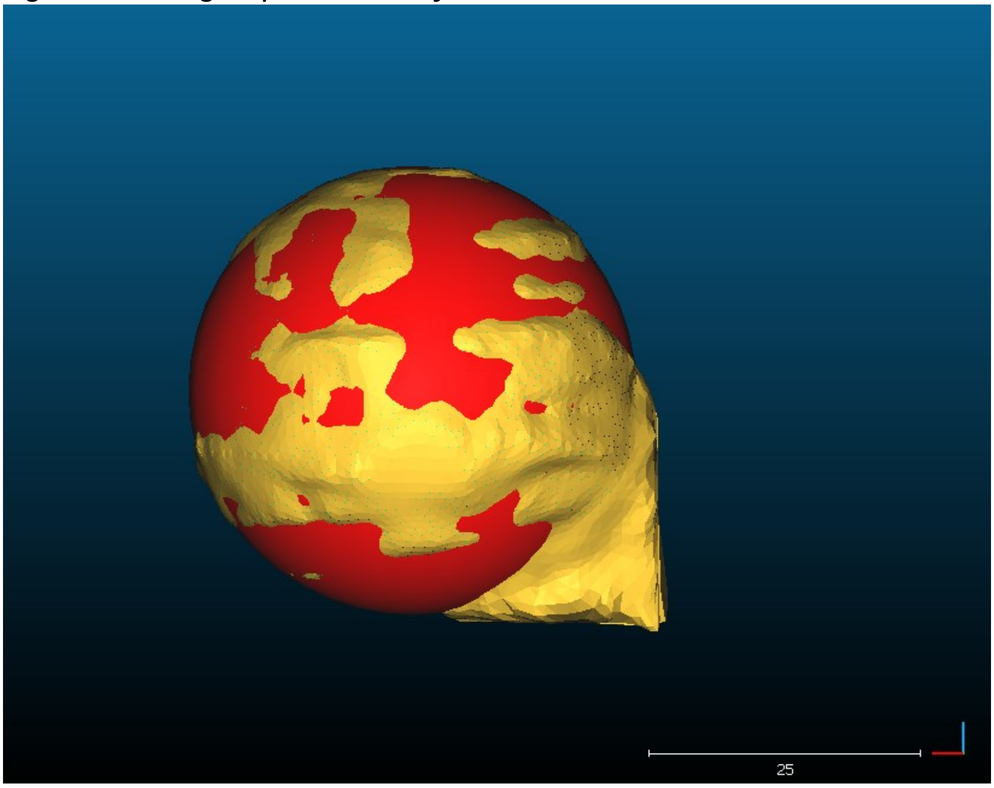

Figure 5: Accuracy of visual localisation of the centre of the femoral head

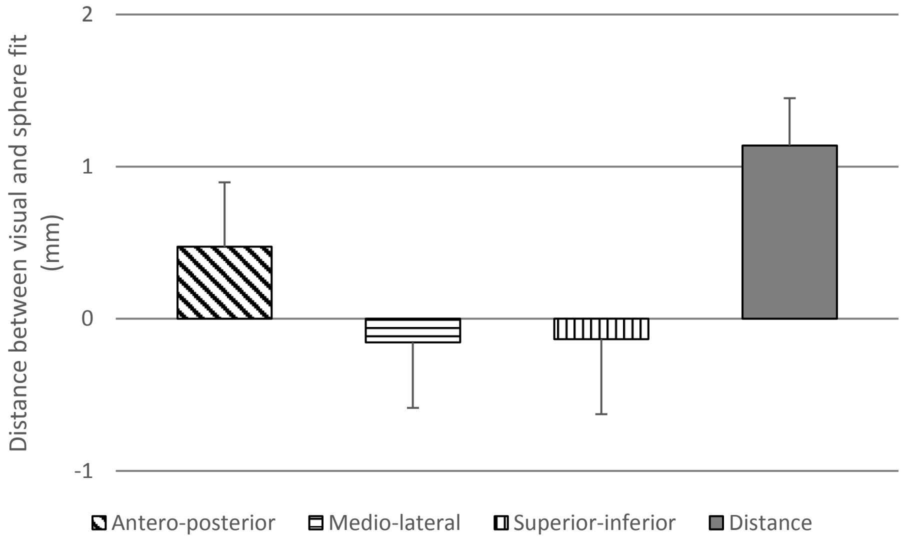

References

1 Schnabel, R., Wahl, R. & Klein, R. Efficient RANSAC for Point-Cloud Shape Detection. *Computer Graphics Forum* **26**, 214--226, (2007).

2 Sangeux, M., Pascoe, J., Graham, H. K., Ramanauskas, F. & Cain, T. Three-dimensional measurement of femoral neck anteversion and neck shaft angle. *J Comput Assist Tomogr* **39**, 83-85, (2015).

Full data set

Headings

**KEY**	Unique number to represent the individual
**AGE**	Age
**GROUP (3)**	Age group with 3 categories: children (1), adolescents (2) and adults (3)
**GROUP (2)**	Age group with 2 categories: children (C) and skeletally matured (A)
**SEX**	Sex: Male or Female
**SIDE**	Side: Right or Left
**Inter ASIS distance (mm)**	Distance between the left and right ASIS
**Clinical Leg Length (mm)**	Clinical leg length, measured from ASIS to medial epicondyle of the knee, then to medial malleola
**Total Pelvic Width (mm)**	Distance between the two most lateral points of the pelvis (edge of the iliac crest)
**Pelvic Depth (mm)**	Distance between the ASIS midpoint and the PSIS midpoint
**Height (mm)**	Height, measured on the CT table
**Weight (kg)**	Weight, measured on the CT table
**HJCx (mm)**	X (antero-posterior) position of the hip joint centre
**HJCy (mm)**	Y (lateral-medial) position of the hip joint centre
**HJCz (mm)**	Z (up-down) position of the hip joint centre
	Origin of the pelvis is set to the mid-point between the Left and right ASIS<br>Transverse plane of the pelvis contain the A/P SIS
**Additional information**	Equations may be derived using total pelvic width and pelvic depth. These equations often performed better than those with inter ASIS distance or clinical leg length. However, the corresponding measurement during gait analysis will be larger because it includes the thickness of adipose tissue.<br><br>We found that height could also be used in place of clinical leg length with equivalent, if not better, accuracy. However, height was measured on the CT table and may not be accurate depending on the posture of the deceased and we chose not to use it.

Data

KEY	AGE	GROUP (3)	GROUP (2)	SEX	SIDE	Inter ASIS distance (mm)	Clinical Leg Length (mm)	Total Pelvic Width (mm)	Pelvic Depth (mm)	Height (mm)	Weight (kg)	HJCx (mm)	HJCy (mm)	HJCz (mm)
1101	5	1	C	M	R	156.1	476.8	169.4	64.9	1040	17	-12.4	48.7	-45.4
1102	5	1	C	M	R	154.3	524.5	173.9	77.4	1000	22	-18.0	53.5	-47.8
1103	5	1	C	M	R	145.9	511.9	161.0	77.3	1050	18	-22.0	51.3	-51.2
1104	5	1	C	M	R	139.7	551.9	166.1	83.6	1160	17	-27.6	55.6	-54.4
1105	5	1	C	M	R	153.1	539.3	178.5	80.9	1160	26	-27.0	54.9	-49.6
1106	9	1	C	M	R	183.7	675.0	200.2	89.7	1380	38	-21.3	66.0	-64.0
1107	6	1	C	M	R	154.6	550.7	172.6	78.7	1130	23	-16.9	53.2	-53.4
1108	5	1	C	M	R	171.0	547.5	191.1	81.2	1170	28	-14.7	59.1	-54.8
1109	9	1	C	M	R	169.1	748.7	200.4	108.5	1470	36	-40.9	65.7	-61.7
1111	6	1	C	M	R	158.7	585.5	189.0	89.8	1190	22	-30.2	58.8	-55.8
1112	9	1	C	M	R	177.2	684.0	202.1	97.5	1350	38	-32.4	61.4	-61.8
1113	5	1	C	M	R	147.2	507.3	168.0	80.0	1040	17	-24.1	50.8	-45.6
1114	8	1	C	M	R	190.9	722.3	216.8	102.0	1410	44	-34.9	66.3	-62.3
1115	6	1	C	M	R	181.8	633.8	200.4	88.5	1300	34	-21.7	61.2	-59.8
1116	5	1	C	M	R	161.0	534.5	180.5	81.3	1110	26	-23.8	57.8	-50.6
1117	6	1	C	M	R	138.2	555.8	162.8	78.7	1120	21	-25.8	55.6	-51.1
1118	8	1	C	M	R	181.1	631.9	197.6	86.9	1290	23	-23.2	62.0	-62.4
1119	11	1	C	M	R	225.3	817.3	260.1	120.3	1700	77	-41.2	76.4	-74.5
1120	5	1	C	M	R	144.3	510.4	162.3	73.1	1070	20	-24.3	53.1	-48.5
1121	5	1	C	M	R	153.6	510.0	177.7	77.1	1100	23	-19.9	55.5	-52.1
1122	5	1	C	M	R	144.3	505.3	163.8	69.9	1050	16	-20.6	48.4	-51.6
1123	9	1	C	M	R	166.8	695.8	193.6	94.3	1330	29	-29.3	57.5	-59.7
1124	10	1	C	M	R	223.0	778.9	247.9	104.9	1520	70	-22.8	70.2	-69.8
1125	10	1	C	M	R	175.5	728.6	208.5	106.9	1490	35	-33.9	69.1	-61.9
1201	5	1	C	F	R	139.7	526.5	159.2	80.7	1110	19	-28.9	53.9	-52.5
1202	11	1	C	F	R	170.9	680.0	198.7	100.7	1350	26	-38.0	64.0	-62.0

1203	11	1	C	F	R	199.8	735.7	229.4	109.8	1480	65	-33.8	66.6	-61.9
1204	11	1	C	F	R	196.6	738.6	225.9	123.2	1400	40	-41.3	78.1	-67.3
1205	11	1	C	F	R	224.5	780.2	240.3	107.7	1490	67	-34.9	75.8	-75.7
1206	7	1	C	F	R	148.7	617.8	175.7	86.0	1210	20	-31.3	54.1	-58.1
1207	10	1	C	F	R	198.0	748.4	223.6	111.0	1470	61	-31.3	66.2	-60.9
1208	9	1	C	F	R	174.2	665.8	200.5	92.5	1320	27	-31.3	59.5	-57.9
1209	9	1	C	F	R	174.9	765.7	199.4	95.6	1560	53	-38.0	60.3	-65.6
1210	8	1	C	F	R	177.0	660.0	201.2	91.7	1350	30	-28.2	60.1	-57.7
1211	8	1	C	F	R	174.7	673.4	204.2	94.1	1310	33	-27.5	59.1	-56.1
1212	8	1	C	F	R	166.8	605.0	184.4	87.7	1210	27	-24.4	59.5	-54.2
1213	7	1	C	F	R	161.1	651.2	183.8	83.6	1230	23	-28.6	53.8	-58.9
2101	17	2	A	M	R	222.5	873.1	264.9	136.2	1720	82	-37.2	80.1	-76.9
2102	18	2	A	M	R	234.2	871.5	273.0	143.2	1680	59	-47.8	79.4	-84.8
2103	19	2	A	M	R	198.1	907.1	264.1	151.7	1780	64	-55.7	87.3	-83.9
2104	16	2	A	M	R	242.0	812.8	277.8	126.4	1710	96	-24.8	82.7	-83.9
2105	16	2	A	M	R	202.1	855.4	231.5	123.5	1660	55	-52.2	76.4	-70.5
2106	19	2	A	M	R	227.8	891.7	272.6	140.1	1730	67	-42.7	80.3	-76.7
2107	19	2	A	M	R	217.2	868.8	255.3	138.4	1650	71	-33.5	84.7	-82.7
2108	19	2	A	M	R	231.3	901.2	261.1	132.1	1690	63	-37.2	76.0	-89.0
2109	16	2	A	M	R	225.6	902.9	287.5	146.9	1700	91	-40.0	80.5	-80.6
2110	17	2	A	M	R	214.4	875.6	271.6	142.6	1700	81	-55.2	82.7	-79.6
2111	18	2	A	M	R	185.2	922.2	240.9	148.3	1760	66	-59.2	86.5	-76.8
2112	16	2	A	M	R	242.9	882.1	272.8	142.8	1720	70	-37.4	82.1	-85.7
2113	17	2	A	M	R	199.7	866.7	265.5	143.5	1730	61	-47.6	84.1	-82.6
2114	19	2	A	M	R	201.5	910.2	265.7	157.9	1780	82	-57.3	86.2	-81.5
2115	17	2	A	M	R	216.6	908.1	250.6	145.5	1700	59	-44.1	82.4	-72.6
2116	16	2	A	M	R	235.5	951.2	270.6	144.0	1730	57	-41.8	78.9	-83.3
2117	17	2	A	M	R	226.2	918.7	264.9	139.2	1800	63	-50.6	82.0	-82.7
2118	18	2	A	M	R	203.0	827.1	266.0	145.4	1630	81	-45.3	81.7	-80.9
2119	19	2	A	M	R	263.4	965.9	293.5	145.8	1860	80	-36.3	86.7	-78.8

2120	18	2	A	M	R	217.4	958.8	265.9	155.3	1800	59	-49.6	82.8	-74.7
2121	19	2	A	M	R	198.8	947.9	243.5	142.2	1760	74	-41.9	80.9	-77.7
2122	17	2	A	M	R	208.7	893.3	257.6	148.3	1710	78	-52.7	85.8	-78.7
2123	19	2	A	M	R	226.1	859.4	270.1	150.3	1720	108	-50.1	81.2	-73.7
2124	17	2	A	M	R	236.3	942.5	275.9	137.7	1700	57	-44.9	86.5	-79.7
2125	19	2	A	M	R	231.7	866.7	259.9	145.1	1700	64	-38.1	77.7	-66.8
2126	18	2	A	M	R	248.2	869.4	278.4	127.0	1660	87	-37.8	76.5	-79.9
2127	19	2	A	M	R	223.3	959.4	275.5	151.5	1820	74	-56.3	87.4	-83.6
2128	19	2	A	M	R	200.0	923.7	218.5	119.3	1700	54	-34.3	68.8	-74.3
2129	18	2	A	M	R	230.9	893.8	270.1	140.2	1670	60	-41.3	81.1	-75.6
2130	18	2	A	M	R	221.7	843.0	261.7	128.6	1640	53	-45.0	80.7	-82.1
2201	18	2	A	F	R	159.1	850.8	214.0	148.4	1630	55	-48.6	79.0	-81.1
2202	17	2	A	F	R	187.6	819.3	222.9	149.9	1620	53	-50.9	82.1	-72.8
2203	18	2	A	F	R	209.9	816.8	255.9	143.9	1580	73	-49.4	86.1	-85.9
2204	19	2	A	F	R	209.9	811.3	251.0	136.8	1540	57	-38.9	78.0	-73.6
2205	19	2	A	F	R	197.5	792.3	226.6	127.0	1540	68	-33.3	78.3	-80.4
2206	17	2	A	F	R	215.7	820.4	255.2	141.9	1610	52	-50.2	83.8	-75.8
2207	16	2	A	F	R	211.6	881.8	259.4	152.8	1690	54	-52.2	81.2	-77.2
2208	17	2	A	F	R	215.5	847.5	268.3	146.3	1730	64	-46.3	87.2	-76.8
2209	17	2	A	F	R	191.2	815.6	240.3	136.1	1610	60	-39.4	82.9	-82.6
2210	19	2	A	F	R	187.4	836.4	239.2	142.6	1580	64	-43.6	78.2	-84.1
2211	18	2	A	F	R	255.7	834.5	288.8	149.3	1640	112	-39.9	86.9	-72.8
2212	18	2	A	F	R	213.9	814.4	250.1	131.7	1610	52	-36.0	85.0	-76.2
2213	16	2	A	F	R	205.5	850.8	254.8	137.4	1680	57	-47.6	85.9	-61.7
2214	16	2	A	F	R	202.2	845.1	238.4	136.0	1650	54	-46.0	80.1	-78.4
2215	16	2	A	F	R	231.5	893.4	265.2	145.4	1710	64	-52.7	88.6	-82.3
2216	19	2	A	F	R	214.7	814.9	263.8	137.6	1650	73	-43.3	82.9	-75.2
2217	16	2	A	F	R	226.2	890.1	266.8	145.9	1570	53	-50.3	81.6	-80.2
2218	18	2	A	F	R	243.5	910.4	284.8	136.2	1690	54	-41.8	90.7	-82.6
2219	18	2	A	F	R	207.8	815.7	255.5	144.1	1650	74	-44.1	82.5	-77.5

2220	18	2	A	F	R	204.8	886.0	262.0	138.4	1660	53	-43.4	83.6	-78.7
2221	17	2	A	F	R	213.1	860.9	257.5	141.8	1660	53	-53.5	88.3	-85.5
2222	17	2	A	F	R	228.5	885.8	260.0	137.1	1640	57	-36.9	84.4	-82.7
2223	18	2	A	F	R	219.6	920.6	277.4	162.4	1730	63	-62.0	81.3	-82.6
2224	19	2	A	F	R	208.8	843.2	245.5	137.2	1640	63	-41.5	77.4	-73.2
2225	17	2	A	F	R	228.0	876.0	274.7	147.6	1720	57	-50.3	85.9	-77.2
2226	19	2	A	F	R	204.2	799.3	244.3	136.9	1550	52	-43.3	81.0	-66.2
2227	17	2	A	F	R	191.2	847.0	247.6	142.8	1630	49	-47.9	78.8	-75.4
2228	17	2	A	F	R	213.3	816.7	271.0	140.1	1600	73	-42.4	80.2	-74.3
2229	19	2	A	F	R	220.7	896.4	267.7	144.1	1700	66	-54.2	87.9	-80.5
2230	16	2	A	F	R	214.0	788.9	246.4	133.4	1500	51	-39.9	75.0	-72.8
3101	39	3	A	M	R	238.4	898.8	274.3	133.0	1750	62	-28.3	80.4	-75.0
3102	25	3	A	M	R	220.2	824.9	257.7	130.8	1640	61	-39.4	82.6	-71.9
3103	29	3	A	M	R	211.8	849.5	260.6	142.2	1650	81	-43.7	84.4	-75.4
3104	33	3	A	M	R	225.9	923.0	270.7	142.1	1760	95	-36.0	86.1	-85.3
3105	31	3	A	M	R	230.6	871.7	272.0	151.7	1770	80	-48.9	95.0	-84.3
3106	25	3	A	M	R	211.0	819.5	261.1	146.5	1640	67	-45.3	81.7	-72.1
3107	37	3	A	M	R	197.0	853.4	255.1	152.5	1670	103	-53.6	86.7	-80.6
3108	36	3	A	M	R	242.4	885.4	291.7	148.6	1700	107	-41.3	80.7	-80.8
3109	35	3	A	M	R	260.9	883.7	296.3	137.1	1710	77	-41.6	88.1	-80.7
3110	28	3	A	M	R	230.0	888.6	270.5	139.7	1730	70	-45.6	83.7	-81.4
3111	31	3	A	M	R	224.8	839.6	259.1	136.1	1660	70	-42.5	82.2	-78.3
3112	31	3	A	M	R	234.3	947.8	260.5	142.4	1780	58	-47.4	88.0	-83.4
3113	40	3	A	M	R	220.1	982.2	270.9	161.6	1890	123	-56.8	97.1	-85.5
3114	28	3	A	M	R	224.3	881.5	260.6	151.5	1700	67	-48.7	90.9	-69.0
3115	34	3	A	M	R	247.3	958.1	282.0	149.6	1840	70	-49.5	96.6	-86.2
3116	35	3	A	M	R	194.4	786.0	223.8	128.0	1550	74	-49.0	75.2	-72.3
3117	30	3	A	M	R	223.0	892.5	263.9	121.2	1700	73	-37.5	81.2	-72.6
3118	31	3	A	M	R	224.2	925.9	284.9	153.2	1750	65	-43.6	95.4	-85.6
3119	26	3	A	M	R	243.3	852.2	277.1	135.9	1710	62	-43.6	87.7	-76.2

3120	32	3	A	M	R	255.5	964.0	296.2	150.5	1840	90	-53.3	94.6	-87.2
3121	38	3	A	M	R	224.3	927.6	262.9	149.6	1740	84	-50.9	89.2	-88.8
3122	25	3	A	M	R	242.5	953.2	288.6	143.6	1840	135	-41.4	94.3	-88.8
3123	27	3	A	M	R	199.8	992.2	261.1	148.1	1840	76	-41.7	86.7	-80.1
3124	25	3	A	M	R	239.8	1045.7	293.1	162.3	1900	78	-52.1	88.0	-88.7
3125	32	3	A	M	R	229.2	864.1	301.7	153.4	1690	79	-54.5	89.1	-80.7
3126	39	3	A	M	R	232.9	896.0	261.4	145.8	1710	93	-37.0	88.7	-75.1
3127	37	3	A	M	R	252.2	993.8	296.9	152.6	1880	121	-51.1	96.9	-91.9
3128	29	3	A	M	R	243.2	955.5	280.4	130.3	1730	77	-41.2	83.7	-77.8
3129	26	3	A	M	R	245.9	941.6	290.0	149.6	1800	86	-38.6	83.6	-81.4
3130	29	3	A	M	R	229.2	937.2	270.3	157.0	1800	88	-45.6	80.3	-82.6
3201	25	3	A	F	R	220.7	905.7	271.3	157.1	1700	107	-59.8	88.9	-88.6
3202	35	3	A	F	R	244.9	827.9	275.4	132.8	1610	84	-46.9	87.4	-80.5
3203	31	3	A	F	R	221.7	855.4	267.3	140.4	1650	52	-46.0	81.7	-79.0
3204	30	3	A	F	R	224.7	799.5	249.1	126.1	1510	44	-43.6	84.3	-62.3
3205	37	3	A	F	R	200.7	802.7	259.3	145.1	1580	67	-49.8	82.4	-73.2
3206	28	3	A	F	R	228.4	806.9	277.8	147.0	1620	59	-43.4	91.4	-71.8
3207	29	3	A	F	R	229.4	875.0	271.3	146.2	1670	68	-42.8	85.9	-82.0
3208	35	3	A	F	R	222.0	776.7	272.7	150.0	1560	109	-44.5	90.2	-76.2
3209	34	3	A	F	R	200.3	877.8	241.4	138.6	1570	52	-44.8	88.2	-73.1
3210	38	3	A	F	R	210.2	875.5	259.2	145.5	1720	69	-44.5	93.8	-91.7
3211	35	3	A	F	R	248.7	820.9	275.2	131.3	1580	94	-27.6	83.4	-80.6
3212	37	3	A	F	R	216.1	904.2	248.5	122.3	1610	56	-40.2	81.1	-73.7
3213	38	3	A	F	R	198.4	820.8	265.2	151.0	1620	89	-39.6	86.7	-68.3
3214	29	3	A	F	R	236.9	823.4	277.0	136.2	1600	57	-40.1	88.3	-71.3
3215	30	3	A	F	R	213.6	806.5	261.1	142.9	1650	97	-47.9	83.7	-74.1
3216	25	3	A	F	R	216.1	758.0	255.9	129.0	1500	51	-37.8	74.0	-64.3
3217	33	3	A	F	R	200.9	870.4	275.2	149.4	1710	76	-53.9	95.1	-83.7
3218	31	3	A	F	R	252.2	832.3	291.9	137.3	1660	72	-37.3	86.4	-75.2
3219	25	3	A	F	R	248.7	893.7	294.5	142.8	1700	79	-46.6	86.5	-76.3

3220	39	3	A	F	R	224.0	897.6	289.1	156.1	1700	87	-58.0	94.6	-78.7
3221	25	3	A	F	R	192.2	796.0	231.6	134.0	1570	67	-44.0	77.7	-75.9
3222	33	3	A	F	R	219.3	776.5	270.4	137.1	1570	50	-40.0	79.0	-73.0
3223	38	3	A	F	R	259.1	877.4	288.4	141.7	1630	58	-44.0	82.6	-74.0
3224	29	3	A	F	R	217.7	820.8	258.7	143.4	1600	53	-37.4	77.7	-68.4
3225	31	3	A	F	R	233.9	911.8	286.9	150.7	1690	72	-46.1	86.4	-75.9
3226	29	3	A	F	R	255.0	879.7	299.0	155.3	1700	65	-51.6	94.2	-85.0
3227	40	3	A	F	R	210.7	802.4	273.0	129.8	1550	85	-46.7	79.5	-78.2
3228	39	3	A	F	R	198.8	849.4	264.0	159.7	1610	83	-49.3	96.6	-73.3
3229	37	3	A	F	R	244.7	938.3	271.3	147.0	1770	111	-45.4	88.7	-76.2
3230	36	3	A	F	R	227.7	820.0	274.4	151.8	1640	61	-43.5	92.0	-71.8
1101	5	1	C	M	L	156.1	475.4	169.4	64.9	1040	17	-13.0	45.3	-43.8
1102	5	1	C	M	L	154.3	528.1	173.9	77.4	1000	22	-18.0	52.0	-47.3
1103	5	1	C	M	L	145.9	509.6	161.0	77.3	1050	18	-21.1	53.1	-52.2
1104	5	1	C	M	L	139.7	560.4	166.1	83.6	1160	17	-28.5	55.2	-54.4
1105	5	1	C	M	L	153.1	537.4	178.5	80.9	1160	26	-27.4	55.3	-49.4
1106	9	1	C	M	L	183.7	664.8	200.2	89.7	1380	38	-20.3	63.6	-63.8
1107	6	1	C	M	L	154.6	516.4	172.6	78.7	1130	23	-16.0	53.3	-53.6
1108	5	1	C	M	L	171.0	551.6	191.1	81.2	1170	28	-15.5	58.7	-54.7
1109	9	1	C	M	L	169.1	752.5	200.4	108.5	1470	36	-41.6	67.9	-62.9
1111	6	1	C	M	L	158.7	583.1	189.0	89.8	1190	22	-31.0	60.2	-50.8
1112	9	1	C	M	L	177.2	727.3	202.1	97.5	1350	38	-33.2	62.0	-61.9
1113	5	1	C	M	L	147.2	501.1	168.0	80.0	1040	17	-25.1	52.0	-45.5
1114	8	1	C	M	L	190.9	710.3	216.8	102.0	1410	44	-35.3	66.9	-62.3
1115	6	1	C	M	L	181.8	630.6	200.4	88.5	1300	34	-17.6	59.4	-59.1
1116	5	1	C	M	L	161.0	535.9	180.5	81.3	1110	26	-23.9	56.5	-53.6
1117	6	1	C	M	L	138.2	558.6	162.8	78.7	1120	21	-28.0	57.5	-51.4
1118	8	1	C	M	L	181.1	665.5	197.6	86.9	1290	23	-21.9	58.6	-62.7
1119	11	1	C	M	L	225.3	817.0	260.1	120.3	1700	77	-42.6	78.7	-74.8
1120	5	1	C	M	L	144.3	508.1	162.3	73.1	1070	20	-20.5	48.9	-49.3

1121	5	1	C	M	L	153.6	510.9	177.7	77.1	1100	23	-19.9	55.6	-51.8
1122	5	1	C	M	L	144.3	508.3	163.8	69.9	1050	16	-25.8	52.0	-49.8
1123	9	1	C	M	L	166.8	699.9	193.6	94.3	1330	29	-31.2	57.5	-59.3
1124	10	1	C	M	L	223.0	790.0	247.9	104.9	1520	70	-24.1	71.2	-68.0
1125	10	1	C	M	L	175.5	729.3	208.5	106.9	1490	35	-31.4	61.0	-61.0
1201	5	1	C	F	L	139.7	522.0	159.2	80.7	1110	19	-28.1	55.2	-51.2
1202	11	1	C	F	L	170.9	674.3	198.7	100.7	1350	26	-37.3	67.6	-61.7
1203	11	1	C	F	L	199.8	734.9	229.4	109.8	1480	65	-31.1	66.1	-63.0
1204	11	1	C	F	L	196.6	734.4	225.9	123.2	1400	40	-39.9	71.4	-68.2
1205	11	1	C	F	L	224.5	779.8	240.3	107.7	1490	67	-34.9	73.2	-73.9
1206	7	1	C	F	L	148.7	610.8	175.7	86.0	1210	20	-31.6	53.7	-55.9
1207	10	1	C	F	L	198.0	757.7	223.6	111.0	1470	61	-31.6	66.6	-57.6
1208	9	1	C	F	L	174.2	663.6	200.5	92.5	1320	27	-31.7	59.9	-58.1
1209	9	1	C	F	L	174.9	767.6	199.4	95.6	1560	53	-36.7	58.3	-66.3
1210	8	1	C	F	L	177.0	697.7	201.2	91.7	1350	30	-28.9	54.7	-55.9
1211	8	1	C	F	L	174.7	670.6	204.2	94.1	1310	33	-27.1	59.1	-55.8
1212	8	1	C	F	L	166.8	606.3	184.4	87.7	1210	27	-22.7	55.5	-55.1
1213	7	1	C	F	L	161.1	651.8	183.8	83.6	1230	23	-29.3	56.1	-57.9
2101	17	2	A	M	L	222.5	874.6	264.9	136.2	1720	82	-33.9	82.9	-77.8
2102	18	2	A	M	L	234.2	868.3	273.0	143.2	1680	59	-43.6	81.5	-86.6
2103	19	2	A	M	L	198.1	908.7	264.1	151.7	1780	64	-56.0	82.6	-82.3
2104	16	2	A	M	L	242.0	826.9	277.8	126.4	1710	96	-29.5	85.3	-83.5
2105	16	2	A	M	L	202.1	850.4	231.5	123.5	1660	55	-51.4	72.5	-68.0
2106	19	2	A	M	L	227.8	907.5	272.6	140.1	1730	67	-44.0	87.3	-78.5
2107	19	2	A	M	L	217.2	870.9	255.3	138.4	1650	71	-36.3	84.7	-82.6
2108	19	2	A	M	L	231.3	908.5	261.1	132.1	1690	63	-39.0	80.9	-87.3
2109	16	2	A	M	L	225.6	900.1	287.5	146.9	1700	91	-38.2	88.2	-83.4
2110	17	2	A	M	L	214.4	876.7	271.6	142.6	1700	81	-54.4	85.0	-79.1
2111	18	2	A	M	L	185.2	923.2	240.9	148.3	1760	66	-58.5	83.4	-76.7
2112	16	2	A	M	L	242.9	885.2	272.8	142.8	1720	70	-40.0	83.9	-87.3

2113	17	2	A	M	L	199.7	875.8	265.5	143.5	1730	61	-49.0	91.3	-85.8
2114	19	2	A	M	L	201.5	917.4	265.7	157.9	1780	82	-55.3	83.1	-83.9
2115	17	2	A	M	L	216.6	890.7	250.6	145.5	1700	59	-44.7	82.5	-71.2
2116	16	2	A	M	L	235.5	949.4	270.6	144.0	1730	57	-44.1	78.3	-82.1
2117	17	2	A	M	L	226.2	917.6	264.9	139.2	1800	63	-50.6	83.8	-83.9
2118	18	2	A	M	L	203.0	831.1	266.0	145.4	1630	81	-48.7	85.9	-80.4
2119	19	2	A	M	L	263.4	964.4	293.5	145.8	1860	80	-36.7	85.3	-79.1
2120	18	2	A	M	L	217.4	956.2	265.9	155.3	1800	59	-51.0	87.1	-70.9
2121	19	2	A	M	L	198.8	955.5	243.5	142.2	1760	74	-46.8	80.5	-78.8
2122	17	2	A	M	L	208.7	890.5	257.6	148.3	1710	78	-49.2	89.0	-75.8
2123	19	2	A	M	L	226.1	848.5	270.1	150.3	1720	108	-51.1	84.0	-75.5
2124	17	2	A	M	L	236.3	957.2	275.9	137.7	1700	57	-46.8	85.2	-79.2
2125	19	2	A	M	L	231.7	876.3	259.9	145.1	1700	64	-42.5	83.4	-67.6
2126	18	2	A	M	L	248.2	860.9	278.4	127.0	1660	87	-40.0	82.8	-79.3
2127	19	2	A	M	L	223.3	963.2	275.5	151.5	1820	74	-50.7	92.6	-87.6
2128	19	2	A	M	L	200.0	939.4	218.5	119.3	1700	54	-33.3	65.9	-75.0
2129	18	2	A	M	L	230.9	909.2	270.1	140.2	1670	60	-41.9	87.1	-78.0
2130	18	2	A	M	L	221.7	842.4	261.7	128.6	1640	53	-43.3	83.6	-83.3
2201	18	2	A	F	L	159.1	852.5	214.0	148.4	1630	55	-51.1	86.0	-80.6
2202	17	2	A	F	L	187.6	819.1	222.9	149.9	1620	53	-47.8	82.4	-72.7
2203	18	2	A	F	L	209.9	831.6	255.9	143.9	1580	73	-51.6	87.0	-86.3
2204	19	2	A	F	L	209.9	804.9	251.0	136.8	1540	57	-41.1	75.0	-70.1
2205	19	2	A	F	L	197.5	786.8	226.6	127.0	1540	68	-32.4	80.0	-77.5
2206	17	2	A	F	L	215.7	806.6	255.2	141.9	1610	52	-46.6	81.2	-75.4
2207	16	2	A	F	L	211.6	880.1	259.4	152.8	1690	54	-52.3	80.6	-75.9
2208	17	2	A	F	L	215.5	849.0	268.3	146.3	1730	64	-44.5	83.2	-76.7
2209	17	2	A	F	L	191.2	809.5	240.3	136.1	1610	60	-43.9	81.0	-76.3
2210	19	2	A	F	L	187.4	829.3	239.2	142.6	1580	64	-46.4	77.6	-84.1
2211	18	2	A	F	L	255.7	837.7	288.8	149.3	1640	112	-39.9	86.1	-76.0
2212	18	2	A	F	L	213.9	823.7	250.1	131.7	1610	52	-38.7	86.2	-77.0

2213	16	2	A	F	L	205.5	855.8	254.8	137.4	1680	57	-47.6	86.8	-64.7
2214	16	2	A	F	L	202.2	847.0	238.4	136.0	1650	54	-49.1	80.5	-76.5
2215	16	2	A	F	L	231.5	894.7	265.2	145.4	1710	64	-52.0	83.6	-83.0
2216	19	2	A	F	L	214.7	820.9	263.8	137.6	1650	73	-46.1	87.2	-77.7
2217	16	2	A	F	L	226.2	894.3	266.8	145.9	1570	53	-49.1	83.1	-81.3
2218	18	2	A	F	L	243.5	911.0	284.8	136.2	1690	54	-33.6	84.3	-84.4
2219	18	2	A	F	L	207.8	816.6	255.5	144.1	1650	74	-40.9	86.7	-80.2
2220	18	2	A	F	L	204.8	880.8	262.0	138.4	1660	53	-40.9	83.3	-78.3
2221	17	2	A	F	L	213.1	856.4	257.5	141.8	1660	53	-49.0	90.1	-87.6
2222	17	2	A	F	L	228.5	889.5	260.0	137.1	1640	57	-34.2	85.0	-84.2
2223	18	2	A	F	L	219.6	925.4	277.4	162.4	1730	63	-61.5	89.0	-85.6
2224	19	2	A	F	L	208.8	852.1	245.5	137.2	1640	63	-40.3	80.7	-76.0
2225	17	2	A	F	L	228.0	875.6	274.7	147.6	1720	57	-48.2	87.4	-80.6
2226	19	2	A	F	L	204.2	814.6	244.3	136.9	1550	52	-41.4	78.2	-67.7
2227	17	2	A	F	L	191.2	841.5	247.6	142.8	1630	49	-51.8	87.0	-75.2
2228	17	2	A	F	L	213.3	819.5	271.0	140.1	1600	73	-47.1	79.1	-73.8
2229	19	2	A	F	L	220.7	906.9	267.7	144.1	1700	66	-54.4	86.9	-82.1
2230	16	2	A	F	L	214.0	781.4	246.4	133.4	1500	51	-44.4	83.1	-73.7
3101	39	3	A	M	L	238.4	903.0	274.3	133.0	1750	62	-30.1	82.3	-75.8
3102	25	3	A	M	L	220.2	829.8	257.7	130.8	1640	61	-35.8	81.8	-72.2
3103	29	3	A	M	L	211.8	842.1	260.6	142.2	1650	81	-38.9	81.4	-76.4
3104	33	3	A	M	L	225.9	914.5	270.7	142.1	1760	95	-41.6	88.3	-80.6
3105	31	3	A	M	L	230.6	877.3	272.0	151.7	1770	80	-48.5	86.5	-83.7
3106	25	3	A	M	L	211.0	820.9	261.1	146.5	1640	67	-46.7	88.9	-73.5
3107	37	3	A	M	L	197.0	845.5	255.1	152.5	1670	103	-53.1	88.0	-81.2
3108	36	3	A	M	L	242.4	882.7	291.7	148.6	1700	107	-42.2	87.0	-78.1
3109	35	3	A	M	L	260.9	890.9	296.3	137.1	1710	77	-43.1	90.5	-81.2
3110	28	3	A	M	L	230.0	892.8	270.5	139.7	1730	70	-47.1	86.7	-82.1
3111	31	3	A	M	L	224.8	841.1	259.1	136.1	1660	70	-43.3	80.6	-79.7
3112	31	3	A	M	L	234.3	958.5	260.5	142.4	1780	58	-44.7	80.7	-82.8

3113	40	3	A	M	L	220.1	993.4	270.9	161.6	1890	123	-61.0	92.7	-88.1
3114	28	3	A	M	L	224.3	906.0	260.6	151.5	1700	67	-47.2	85.3	-71.4
3115	34	3	A	M	L	247.3	968.0	282.0	149.6	1840	70	-56.6	92.9	-87.5
3116	35	3	A	M	L	194.4	784.1	223.8	128.0	1550	74	-47.3	74.9	-72.6
3117	30	3	A	M	L	223.0	896.9	263.9	121.2	1700	73	-37.3	75.9	-72.1
3118	31	3	A	M	L	224.2	924.6	284.9	153.2	1750	65	-42.3	91.8	-86.6
3119	26	3	A	M	L	243.3	844.7	277.1	135.9	1710	62	-42.9	84.0	-76.8
3120	32	3	A	M	L	255.5	965.8	296.2	150.5	1840	90	-51.9	92.1	-85.0
3121	38	3	A	M	L	224.3	925.1	262.9	149.6	1740	84	-53.3	84.9	-86.8
3122	25	3	A	M	L	242.5	965.9	288.6	143.6	1840	135	-42.9	90.2	-89.6
3123	27	3	A	M	L	199.8	998.7	261.1	148.1	1840	76	-44.9	86.3	-79.8
3124	25	3	A	M	L	239.8	1065.2	293.1	162.3	1900	78	-54.2	88.4	-88.4
3125	32	3	A	M	L	229.2	863.1	301.7	153.4	1690	79	-52.6	91.3	-80.1
3126	39	3	A	M	L	232.9	907.9	261.4	145.8	1710	93	-37.9	85.9	-76.5
3127	37	3	A	M	L	252.2	997.3	296.9	152.6	1880	121	-51.5	90.4	-91.5
3128	29	3	A	M	L	243.2	954.1	280.4	130.3	1730	77	-38.1	83.2	-78.3
3129	26	3	A	M	L	245.9	952.4	290.0	149.6	1800	86	-42.0	88.7	-82.8
3130	29	3	A	M	L	229.2	944.1	270.3	157.0	1800	88	-50.4	82.9	-81.9
3201	25	3	A	F	L	220.7	907.0	271.3	157.1	1700	107	-53.7	99.0	-89.8
3202	35	3	A	F	L	244.9	834.2	275.4	132.8	1610	84	-45.0	87.8	-79.6
3203	31	3	A	F	L	221.7	857.7	267.3	140.4	1650	52	-42.6	83.5	-81.4
3204	30	3	A	F	L	224.7	803.3	249.1	126.1	1510	44	-41.2	85.4	-65.3
3205	37	3	A	F	L	200.7	806.4	259.3	145.1	1580	67	-51.9	87.2	-72.1
3206	28	3	A	F	L	228.4	813.7	277.8	147.0	1620	59	-45.3	88.8	-71.1
3207	29	3	A	F	L	229.4	880.1	271.3	146.2	1670	68	-43.9	88.5	-82.4
3208	35	3	A	F	L	222.0	779.1	272.7	150.0	1560	109	-47.4	90.1	-77.9
3209	34	3	A	F	L	200.3	871.4	241.4	138.6	1570	52	-44.4	78.3	-72.7
3210	38	3	A	F	L	210.2	879.4	259.2	145.5	1720	69	-38.2	90.8	-94.0
3211	35	3	A	F	L	248.7	821.5	275.2	131.3	1580	94	-27.5	88.0	-80.0
3212	37	3	A	F	L	216.1	900.0	248.5	122.3	1610	56	-37.7	76.5	-73.4

3213	38	3	A	F	L	198.4	814.6	265.2	151.0	1620	89	-43.3	88.1	-71.1
3214	29	3	A	F	L	236.9	824.8	277.0	136.2	1600	57	-41.7	87.7	-70.9
3215	30	3	A	F	L	213.6	808.0	261.1	142.9	1650	97	-45.0	89.5	-77.0
3216	25	3	A	F	L	216.1	762.8	255.9	129.0	1500	51	-36.6	83.7	-69.6
3217	33	3	A	F	L	200.9	874.0	275.2	149.4	1710	76	-56.3	88.4	-86.7
3218	31	3	A	F	L	252.2	835.0	291.9	137.3	1660	72	-38.4	85.6	-73.1
3219	25	3	A	F	L	248.7	900.7	294.5	142.8	1700	79	-44.7	91.7	-78.2
3220	39	3	A	F	L	224.0	899.6	289.1	156.1	1700	87	-59.6	91.3	-78.1
3221	25	3	A	F	L	192.2	800.0	231.6	134.0	1570	67	-44.0	79.0	-79.1
3222	33	3	A	F	L	219.3	784.0	270.4	137.1	1570	50	-39.2	80.5	-72.6
3223	38	3	A	F	L	259.1	878.8	288.4	141.7	1630	58	-40.3	84.2	-74.9
3224	29	3	A	F	L	217.7	826.3	258.7	143.4	1600	53	-38.3	84.4	-68.7
3225	31	3	A	F	L	233.9	920.6	286.9	150.7	1690	72	-49.5	85.9	-79.6
3226	29	3	A	F	L	255.0	871.6	299.0	155.3	1700	65	-49.1	101.0	-86.2
3227	40	3	A	F	L	210.7	798.9	273.0	129.8	1550	85	-46.6	79.5	-79.0
3228	39	3	A	F	L	198.8	852.0	264.0	159.7	1610	83	-54.1	83.2	-77.0
3229	37	3	A	F	L	244.7	944.3	271.3	147.0	1770	111	-46.4	88.5	-76.7
3230	36	3	A	F	L	227.7	823.4	274.4	151.8	1640	61	-42.6	89.8	-70.6
